# Vaccination and COVID-19: impact on long-COVID

**DOI:** 10.3389/fimmu.2025.1686572

**Published:** 2025-11-19

**Authors:** Gustavo N. Guimarães, Natália S. Brunetti, Dieila G. De Lima, José Luiz Proenca-Modena, Alessandro S. Farias

**Affiliations:** 1 Autoimmune Research Laboratory, Department of Genetics, Microbiology and Immunology, Institute of Biology, University of Campinas, Campinas, SP, Brazil; 2 Life Sciences Core Facility (LaCTAD), University of Campinas, Campinas, SP, Brazil; 3 Laboratory of Emerging Viruses, Department of Genetics, Microbiology and Immunology, Institute of Biology, University of Campinas, Campinas, SP, Brazil

**Keywords:** COVID-19, Long-COVID, post-COVID syndrome, SARS-CoV-2, vaccine, immunology

## Abstract

Long- and post-COVID-19 syndromes have emerged as a significant global health challenge, with millions of individuals experiencing persistent or the development of new symptoms after a long period of an initial SARS-CoV-2 infection. These symptoms are multisystemic and may indicate changes in the respiratory, neurological, cardiovascular and gastrointestinal systems, in addition to prolonged fatigue. Vaccination has played a crucial role in reducing severe disease and mortality, but the impact of the different vaccine combinations on the development and resolution of Long COVID remains a topic of debate. This review synthesizes current evidence on how different vaccine platforms, dosing strategies and booster doses influence the immunological response, protection, incidence, severity, and persistence of Long COVID symptoms. We discuss key immunological mechanisms by which vaccination may modulate and protect post-COVID syndrome outcomes, including its effects on viral clearance, immune response reprogramming, inflammation, and autoimmunity, seeking to combat misinformation and concepts spread by fake news. The review also highlights controversies and research gaps, such as variability in vaccine response among different populations and the role in the selection of more transmissible and virulent SARS-CoV-2 variants, as well as the potential differences between vaccine-induced and infection-induced immunity, and the role of pre-existing immune conditions in this scenario.

## 1 Introduction

**Figure 1:**
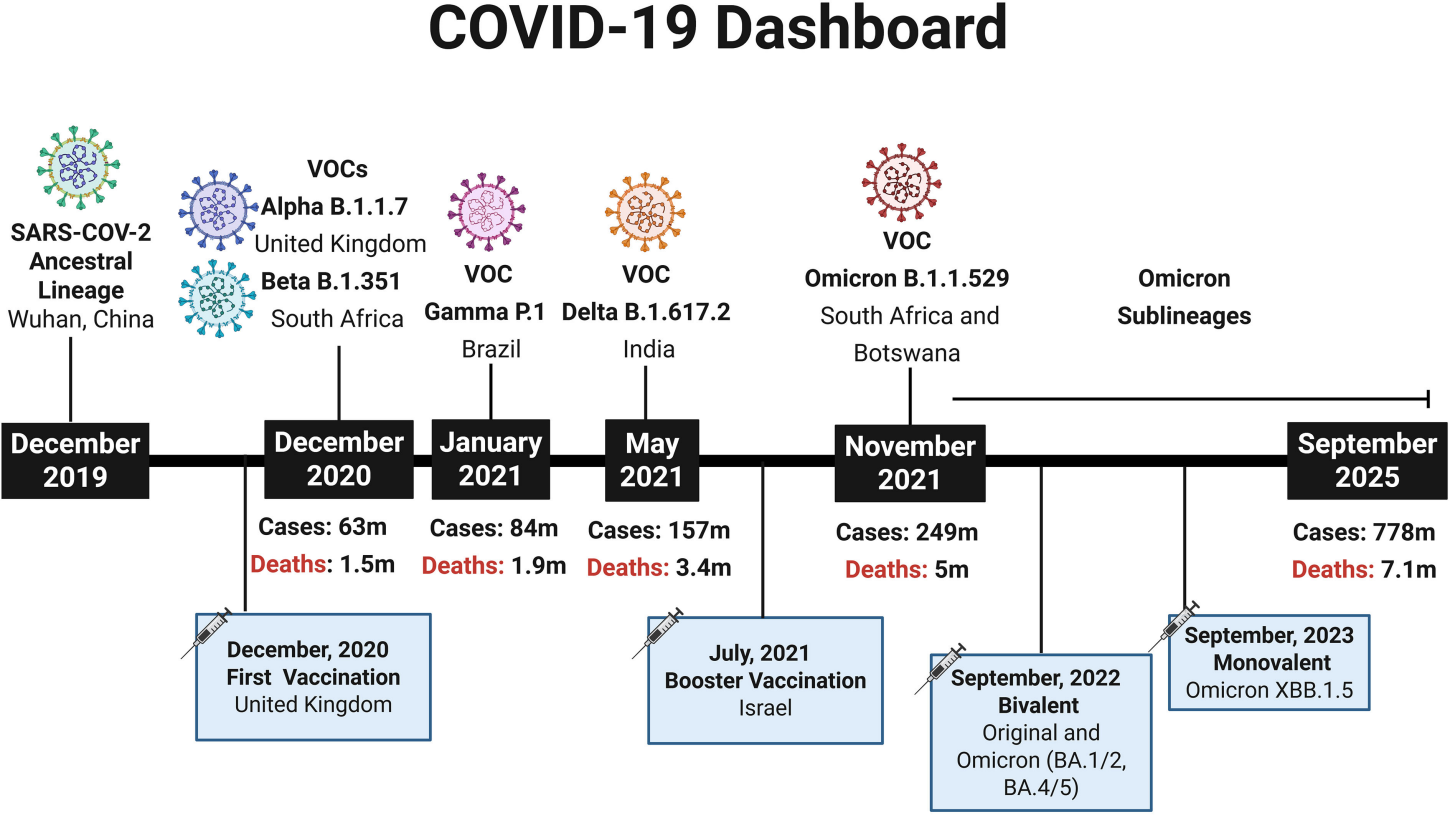
COVID-19 Dashboard summarizing key milestones of the pandemic. Timeline depicting the emergence of the SARS-CoV-2 ancestral lineage and major Variants of Concern (VOCs) according to WHO designations. The figure also indicates the rollout of the first COVID-19 vaccination (December 2020, United Kingdom), the first booster campaigns (July 2021, Israel), the introduction of bivalent vaccine (Original and Omicron BA.1/2, BA4/5) (2022) and the Updated monovalent Omicron vaccine XBB.1.5 (2023). At each highlighted time point, the cumulative numbers of global COVID-19 cases and deaths are shown, based on WHO data (7), providing a broad epidemiological perspective from the initial outbreak in December 2019 to September 2025. Created using Biorender: https://BioRender.com/8fyi7on.

In late December 2019, the World Health Organization (WHO) was notified of an outbreak of pneumonia in Wuhan, China (1), and by early January 2020 a novel coronavirus, later named SARS-CoV-2 by the ICTV and the disease it causes designated COVID-19 by the WHO, was identified as the causative agent (2–4). The rapid global spread of the virus led the WHO to declare a pandemic in March 2020 (5), resulting in major public health measures and an unprecedented mobilization of scientific efforts to develop vaccines. Remarkably, within one year of the first reported cases, the first COVID-19 vaccination outside clinical trials was administered in the UK, amid over 65.8 million confirmed cases and 1.5 million deaths worldwide (6) (**Figure 1**). Given that COVID-19 was a novel disease, the knowledge about the nature of the protective immune response for different population groups was limited, there was uncertainty which vaccine strategies would have more success (8, 9). Therefore, a hallmark of the COVID-19 pandemic was the variety of technology platforms applied to vaccine development against SARS-CoV-2, including inactivated vaccines, adenovirus-vectored vaccines and mRNA vaccines (10, 11).

Since SARS-CoV-2 is an RNA virus, it naturally accumulates mutations at a high rate, driving viral evolution that can enhance replication, transmissibility, and immune evasion (12). Variants with significant impacts on disease severity or reduced vaccine and treatment efficacy are dynamically classified as variants of concern (VOCs) (13). The emergence of VOCs has raised concerns regarding the effectiveness of vaccine-induced immunity, particularly for mRNA and vector-based vaccines, which were designed to express the spike glycoprotein encoded by the original reference strain. As a result, booster doses and updating vaccines were globally recommended, especially for vulnerable groups (14, 15).

Despite overcoming the acute phase of COVID-19, a considerable number of individuals experience the persistence of some initial symptoms, resurgence of previously resolved symptoms, or even the onset of novel symptoms, a condition that has come to be known, depending on its time span, as Long COVID (LC), or Post-Acute Sequelae of COVID-19 (PASC), 4 weeks to three months after COVID-19; and Post-COVID Condition (PCC), three months to years after the disease (16–18). Long COVID is characterized by a broad spectrum of symptoms, including fatigue, cognitive dysfunction, dyspnea, neuropsychiatric disturbances, and multisystemic involvement affecting the respiratory, neurological, cardiovascular, and gastrointestinal systems (19, 20). Although the mechanisms underlying this condition remain incompletely understood, several pathophysiological models have been proposed. These include the persistence of infectious particles or viral fragments (“viral ghosts”) in tissue reservoirs, tissue damage resulting from exacerbated inflammation during a severe acute phase of infection, autoimmunity triggered by the infection, and even reactivation of latent viruses (21, 22).

Alterations in immune responses associated with viral persistence and severe COVID-19 are at least partially triggered by the infection of CD4^+^ T helper cells by SARS-CoV-2, as previously reported (23), leading to T cell death or dysfunctional T cells resulting from inflammatory cytokine storm. Thus, CD4-mediated infection of helper T cells by SARS-CoV-2 may underlie the deficient immune responses observed in some patients with COVID-19 (23). However, how long these alterations in T cell function persist *in vivo*, and whether they exert long-term impacts on adaptive immunity, remains to be determined, particularly considering that, from an evolutionary perspective, the infection of CD4^+^ T cells represents an effective immune evasion strategy employed by viruses (24).

Although the worldwide mass vaccination had played a pivotal role in reducing SARS-CoV-2 infection rates, hospitalizations, and mortality, emphasizing on vaccine effectiveness in COVID-19 prevention or reducing its severity (25), their effect on preventing long COVID is not yet fully understood. Evidence from studies in the general adult population indicates that vaccination confers some degree of protection against the development of long COVID (26). It has been hypothesized that vaccination could confer protective effects against LC through multiple immunological mechanisms, especially reduction of viral burden and limitation of viral reservoir formation, attenuation of severe acute outcomes of SARS-CoV-2 infection and reprogramming of immune responses (27, 28).

Nonetheless, findings remain heterogeneous across studies, often influenced by vaccine platform (mRNA, viral vector, inactivated virus), number of doses, booster regimens, timing of vaccination relative to infection, and host-related factors such as comorbidities, socioeconomic conditions and immune status. Also, the clinical course of SARS-CoV-2 infection and the long-term effects of COVID-19 are also influenced by the evolution of the virus and the emergence of new variants, highlighting ongoing concern about the role of widespread vaccination in shaping viral evolution, potentially contributing to the emergence of more transmissible or immune-evasive SARS-CoV-2 variants (7). Therefore, it is still uncertain how vaccine-induced immunity compares to infection-induced immunity in modulating long COVID risk. This review aims to synthesize current evidence on the role of COVID-19 vaccination in the prevention and modulation of long COVID/post-COVID syndromes. Here we explore the immunological mechanisms underlying vaccine effects, the influence of different vaccine platforms and dosing strategies, the timing of vaccination in relation to SARS-CoV-2 infection, and responses among specific populations.

## 2 COVID-19

### 2.1 Viral biology of SARS-CoV-2

SARS-CoV-2 is a positive-sense single-stranded RNA virus (+ssRNA) and has a size around 30 kilobases (kb). Its genomic structure consists of six major ORFs (Open Reading Frames), which encode non-structural proteins related to transcription and replication (ORF1a/ORF1b) and structural proteins such as Spike (S), Envelope (E), Membrane (M) and Nucleocapsid (N). The RNA genome is surrounded by a helical protein coat, forming the viral nucleocapsid. This nucleocapsid is embedded in a lipoprotein envelope composed of phospholipid molecules and structural proteins (M, E and S) inserted in the lipid bilayer (29).

**Figure 2:**
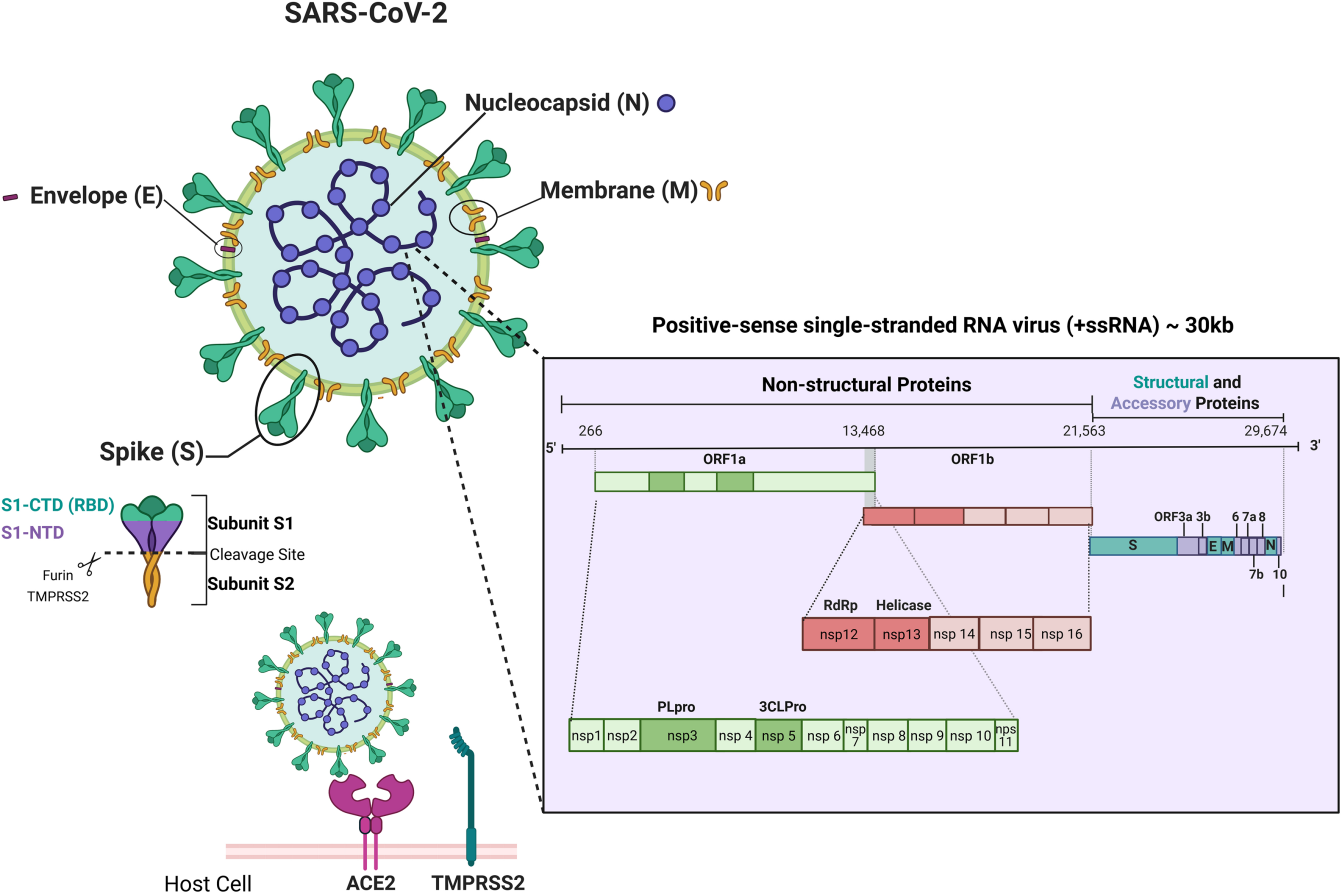
Schematic representation of SARS-CoV-2 structure, genome and main host-cell entry mechanism. Schematic representation of the SARS-CoV-2 virion showing its four main structural proteins — Spike (S), Envelope (E), Membrane (M), and Nucleocapsid (N) — and the viral positive-sense single-stranded RNA genome (~30 kb). The genomic map highlights ORF1a/1b, encoding the non-structural proteins (nsp1–nsp16) involved in replication and transcription (including PLpro, 3CLpro, RdRp, and Helicase), followed by genes for structural and accessory proteins (S, E, M, N, ORF3a, 6, 7a/b, 8, 10). The figure also illustrates the Spike (S) subunits (S1-NTD, S1-CTD/RBD, S2) and its interaction with ACE2 and TMPRSS2 during host-cell entry. Created using Biorender: https://BioRender.com/4xtisqv.

Spike glycoproteins, present on the entire viral surface forming the characteristic coronavirus spikes, consist of two non-covalently associated subunits, each one with a different role in the infection process. While the S1 subunit is responsible for recognizing and binding to cell receptors, the S2 subunit is responsible for the subsequent fusion of cell and viral membranes (30). The S1 subunit contains a portion called the receptor binding domain (RBD), which is capable of binding to the host cell receptor, the angiotensin-converting enzyme 2 (ACE2, Angiotensin-Converting Enzyme 2) (31, 32) (**Figure 2**).

The binding of Spike to the ACE2 receptor followed by activation promoted by a host cell protease, TMPRSS2 (transmembrane serine protease type II), allows the virus entry into the host cell. The activity of other host proteases, such as furin, also contributes to Spike priming and acts synergistically with TMPRSS2 to enhance viral entry and infectivity (33). Inside the cell, SARS-CoV-2 genome is immediately translated, and the viral RNA-dependent RNA polymerase is used to replicate its genome (34), installing an infectious process that triggers immune responses in the host. Although in most cases, the recruited cells clear the infection and the patients recover, the rapid replication of SARS-CoV-2 can trigger a strong and unregulated immune response. This response leads to a phenomenon known as cytokine storm, an overproduction of pro-inflammatory cytokines that causes the recruitment of inflammatory cells to the lungs, causing tissue damage and consequently, the acute respiratory distress syndrome (ARDS), considered the main cause of death in patients with COVID-19 (35–37).

Although ACE2 is widely recognized as the canonical entry receptor for SARS-CoV-2, several molecules have been suggested to serve as alternative receptors or co-factors that may facilitate viral entry. Examples include C-type lectins (such as DC-SIGN and L-SIGN), phosphatidylserine receptors like TIM-1 and TIM-4, the receptor tyrosine kinase AXL, and the transmembrane protein CD147 (38–40).

Notably, Neuropilin-1 (NRP1) has emerged as a potential facilitator of SARS-CoV-2 entry in cells with low ACE2 expression, such as respiratory and olfactory epithelial cells (41). Furthermore, studies show that NRP1 serves as the principal receptor mediating cell entry in astrocytes, the main site of infection and possibly, replication, of SARS-CoV-2 in the brain of COVID-19 patients (42, 43). As astrocytes support neuronal functions, their infection may underlie the neurocognitive and neuropsychiatric symptoms reported in some patients (42).

Also, evidence indicates that SARS-CoV-2 can infect and replicate in lymphocytes, despite their low levels of ACE2 expression (44, 45), which could explain lymphocytopenia and dysregulated inflammatory response in severe COVID-19 patients. The selective infection targeting CD4^+^ T helper cells and the high-affinity interaction between the viral spike RBD and the N-terminal domain of the CD4, assessed by molecular docking and dynamic simulations, and supported by co-immunoprecipitation and fluorescence anisotropy assays, identifies CD4 as a critical cofactor that facilitates viral attachment and promotes SARS-CoV-2 internalization in these cells (23).

Understanding the immune responses to a pathogen’s infection is crucial to know the pathogenesis of the disease, and as a basis for therapeutic applications and vaccine development. This becomes a major challenge in the context of an emerging disease, such as COVID-19, where studies are underway in laboratories around the world while new data are generated (9).

### 2.2 Immunology of COVID-19

#### 2.2.1 Innate immune response

The innate immune response is the first line of defense against infections, comprising natural barriers, immune cells (macrophages, dendritic cells, neutrophils, NK cells), soluble mediators (cytokines, chemokines, natural antibodies), and the complement system. While it may not always eliminate the pathogen, it delays disease progression and supports adaptive immunity. Its activation depends on the recognition of Pathogen-Associated Molecular Patterns (PAMPs) by Pattern Recognition Receptors (PRRs), which are located on cellular or endosomal membranes (46). The detection of PAMPs by PRRs leads to an increase in the production of cytokines and chemokines, responsible for signaling the infectious process. PRRs also detect Damage-Associated Molecular Patterns (DAMPs) generated during infection (47).

During positive-strand RNA viral genome replication occurs the formation of double-stranded RNA (dsRNA), intermediary structures of replication, which are recognized by PRRs, resulting in increased production of type I interferon (IFN-I) (48–50). The production of type I IFNs (mainly IFN-α and IFN-β) plays a central role in the antiviral response, inducing the activation of natural killer (NK) cells and the maturation of antigen-presenting cells (APCs), such as dendritic cells (DCs) and macrophages (51). Upon contact with pathogens, APCs process and associate the viral antigens with Major Histocompatibility Complex (MHC) molecules on the cell surface. Viral intracellular antigens are associated with MHC class I and presented to cytotoxic T lymphocytes (CTLs), also called CD8+ T cells. Whereas virus-infected cells can be cross-presented to T helper lymphocytes (Th), also called CD4+ T cells, via MHC class II. The increased production of IFN-I and the activation of APCs provide an important link between innate and adaptive immune responses (52).

It has been reported that SARS-CoV-2 infection suppresses the innate immunity, reducing the number and maturation of DCs, inhibiting the IFN-I-mediated antiviral response, which can lead to a latent T cell response in patients with COVID-19 (53). This ability may explain the prolonged incubation or pre-symptomatic period of 2–12 days for SARS-CoV-2 compared to the 1–4 days for influenza (10). Furthermore, SARS-CoV-2 can infect monocytes, which certainly compromises the immune response to the virus (54).

Another PRR that has gained prominence in the context of long COVID-19 is Toll-like receptor 4 (TLR4), which plays an essential role in the antibacterial response by detecting the lipopolysaccharide (LPS) molecule of Gram-negative bacteria but is also involved in viral recognition (55). The SARS-CoV-2 Spike protein interacts strongly with TLR4, leading to the activation of this receptor and triggering signaling that increases ACE2 expression on the cell surface, facilitating viral entry into cells that express little ACE2, such as lung cells, in addition to causing hyperinflammation in patients (56). In mice, it has been shown that TLR4 activation by Spike can lead to long-term cognitive dysfunction (57).

#### 2.2.2 Adaptive immune response

The adaptive immune response is a combined action between the cellular response, mediated by T cells, and the humoral response, mediated by B cells. While the presentation of viral antigens associated with MHC-I induces CD8+ T cells to screen and kill all virus-infected or modified cells by secreting cytotoxic granules containing granzymes and perforins, the presentation of viral antigens associated with MHC-II induces the differentiation of CD4+ T cells or T helper cells (Th) into several subpopulations.

Th1 lymphocytes subpopulation secrete interleukins 2 and 12 (IL-2 and IL-12), IFN-I, and Tumor Necrosis Factor-α (TNF-α), promoting cellular immunity, whereas Th2 lymphocytes produce Interleukins (IL-4, IL-5, IL-6, IL-10, and IL-13), stimulating humoral responses through B cell activation and antibody production. The cytokine profile of T helper cells drive the immune response toward cellular or humoral pathways, which depends on the virus and its interaction with the immune system. Understanding these specific mechanisms is essential for effective vaccine development (58).

Although both cellular and humoral responses to SARS-CoV-2 appear within a week of symptom onset (9), studies found that individuals recovering from mild cases retained cell-mediated immunity even when antibody responses were no longer detectable (59, 60). Also, a study with SARS-CoV demonstrated that virus-specific memory CD8+ T cells persisted for up to 6 years post-infection, whereas memory B cells and specific antibodies were undetectable (61). On the other hand, we reported in a previous study, that SARS-CoV-2 may use a CD4 molecule to infect helper T cells, leading to increased expression of the anti-inflammatory cytokine IL-10, which is associated with viral persistence and disease severity. Thus, CD4-mediated infection of helper T cells by SARS-CoV-2 may explain a deficient immune response in some patients with COVID-19 (23).

The activation of the humoral immunity is mediated by immunoglobulins (Igs), popularly known as antibodies. In general, specific antibody levels in blood plasma correlate with the stage of infection and degree of protection, and the IgM detection indicates acute infection, while isolated IgG may reflect chronic infection or immunity acquired during convalescence and after recovery. Thus, the detection of circulating antibody levels is one of the correlates of protection and immunological memory against determined viral infection (58). It has been demonstrated that SARS-CoV-2- specific IgM and IgG antibodies are detectable within 1–2 weeks of symptom onset in most infected individuals (62) and begin to decline within 8 weeks (63).

One of the biological actions performed by IgM, IgG and IgA antibodies in the antiviral response is neutralization, in which antibodies bind to specific regions of the virus, preventing its interaction with cell receptors and neutralizing its infectivity (64). Elevated levels of neutralizing antibodies to the Wuhan lineage have been observed in convalescent individuals (63) and appear to offer some benefit in treatment studies with convalescent plasma, previously used successfully in the treatment of SARS-CoV (65). Among a wide variety of antigenic determinants capable of inducing the production of high titers of neutralizing antibodies, viral surface proteins seem to be the best viral antigenic targets, due to their location, accessibility and function (66). The spike protein S1 subunit, through receptor binding domain (RBD), is the primary target for SARS-CoV-2 neutralizing antibodies (66). Antibodies that bind to RBD block its interaction with ACE2, preventing the virus from fixing to the host cell (10). Furthermore, this domain also contains epitopes for T cell responses (67).

Other important functions in combating viral infections are performed by the Fc portion of antibodies, including complement system fixation, opsonization, phagocytosis, and cellular cytotoxicity (68). Functions such as antibody-dependent cellular phagocytosis (ADCP) and cellular cytotoxicity (ADCC) are still being explored in the context of SARS-CoV-2. In ADCP, phagocytic cells such as macrophages recognize the constant region of the antibody, leading to phagocytosis and elimination of infected cells (69). In ADCC, this process is carried out mainly by natural killer cells that recognize infected cells coated with IgG and secrete cytotoxic granules, leading to cell death (70). Studies have observed that ADCC by NK cells is triggered primarily by non-spike antigens and that hybrid immunization (vaccinated convalescent individuals) can generate antibodies that enhance this activity and provide higher protection to the host against virus variants (71, 72). Regarding the protection generated by antibodies, we can also mention neonatal immunity provided by the transport of maternal IgG to the fetus through the placenta and IgA through breastfeeding (73, 74).

Coronavirus-specific T cells are important in clearing the virus and controlling disease progression and should be considered in vaccine strategies (10, 47). It has been shown that the SARS-CoV-2-induced T helper cells phenotype is associated with the severity of COVID-19 cases (75). Elevated levels of specific T cells have been observed in convalescent individuals, with a higher number of memory CD8+ T cells in the respiratory tract of mild cases compared to severe cases of COVID-19 (59, 63). Other studies have shown a higher proportion of IFNγ-producing Th1 cells in patients with mild and moderate disease, including children, than in patients with severe disease, correlating with viral clearance (76–78). In severe cases of the disease, the immunological imbalance during the acute phase triggers an increase in the production of the cytokines IL-6, IL-10 and IL-8, associated with a Th2 profile, in addition to an exhaustion of CD8+ T lymphocytes through the expression of TIM-3 and PD-1 (79, 80). Regarding SARS-CoV-2-specific T cells generated by natural infection and vaccination, there are mostly central memory and effector memory CD4+ T cells and effector memory and terminally differentiated effector CD8+ cells (81, 82). In children, it has been shown that early immune memory responses seem to be dominated by nucleocapsid-specific CD8+ T cells and antibodies, with a correlation between anti-N antibodies and TNF-α–producing memory CD8+ T cells suggesting a distinctive CD8–B cell crosstalk not typically observed in adults (78). These differences in immune profiles highlight the need for pediatric-specific vaccination strategies that leverage the strengths of children’s cellular immunity.

#### 2.2.3 SARS-CoV-2 mechanisms of immune evasion

SARS-CoV-2 evades both innate and adaptive immunity through multiple mechanisms involving nonstructural proteins (NSPs). NSP1 binds to the 40S ribosomal subunit, blocking host mRNA entry and promoting its degradation, thereby inhibiting type I IFN expression and favoring the translation of viral mRNAs (83, 84). NSP14 and NSP16 help camouflage viral mRNA to resemble host mRNA: NSP14 adds a 7-methylguanosine cap at the 5’ end to evade RIG-1 detection (85), while NSP16 methylates the 2’-hydroxyl of the first ribose to escape MDA-5 recognition (86). These modifications suppress IFN regulatory factors and reduce type I IFN production. Additionally, NSP3 and NSP4 induce double-membrane vesicles (DMVs) from the endoplasmic reticulum, creating protected compartments for viral replication and protein export (87). Infected individuals with moderate to severe COVID-19 also exhibit reduced plasmacytoid dendritic cells (pDCs), key producers of type I IFN critical for viral clearance (88).

Severe COVID-19 cases are associated with an excessive immune response in the lungs, particularly in susceptible individuals, such as those carrying mutations in genes involved in IFN-I production (89) and those with circulating autoantibodies against IFN-I (90). In this scenario, both cellular and humoral immunity become impaired. A reduced antiviral state promotes intense inflammation with overproduction of cytokines such as IL-6, TNF-α, and CXCL10, triggering a cytokine storm. This inflammatory response increases recruitment of myeloid cells (macrophages and neutrophils) and reduces lymphoid cells (NK cells and lymphocytes), leading to complement activation, coagulation, NETosis, and tissue damage, which can be fatal (91). Failure to control the initial infection results in lymph node lymphopenia, CD8+ T cell exhaustion (92), germinal center collapse, impaired high-affinity antibody production, and deficient memory cell formation (93), also contributing to the emergence of new viral variants.

Regarding the mechanisms of escape from the adaptive immune response by SARS-CoV-2, we have evasion via neutralizing antibodies through the emergence of mutations, which mainly affect the receptor-binding domain (RBD) of the Spike surface protein and antigenic target of most vaccines developed and applied to the world population. These mutations alter the protein in such a way as to prevent recognition and binding of antibodies, consequently allowing host cells to be infected. Furthermore, mutations in key epitopes for binding to the MHC compromise other processes of antigenic destruction, such as complement fixation, phagocytosis and antibody-dependent cellular cytotoxicity, and the generation of long-term memory cells (94).

## 3 Long- and post-COVID-19 conditions

When symptomatic, SARS-CoV-2 infection is usually associated with an acute illness, characterized by symptoms such as dyspnea, fever, cough, accounting for about 70% of cases, pharyngitis, nausea, anorexia, anosmia, dysgeusia, cephalgia (34%), malaise, myalgia (36%), and diarrhea (12%) (95–97). Severe COVID-19, involving significant respiratory distress and cytokine storm, usually appears in between 0.3 and 3% of infected patients and is associated with lymphopenia (98), defect in Th1 immunity (92–99) and T cell depletion in lymphoid tissues (93). Other clinical manifestations, such as multisystemic inflammatory syndrome (MIS) and long COVID-19, are also frequent and impact the lives of infected people. MIS is mainly characterized by systemic hyperinflammation, which is difficult to discern from acute biphasic COVID-19 and post-acute sequelae of SARS-CoV-2 infection. MIS is presented after 4 weeks of acute COVID-19 with findings similar to Kawasaki disease (KD) such as fever (>38 °C), rash, conjunctivitis, peripheral edema, lower extremity and abdominal pain, vomiting, and diarrhea (100, 101). Recent advances in findings linked MIS-C to TGFβ overproduction, causing Epstein-Barr virus (EBV) reactivation and hyperinflammation due to an impaired T cell cytotoxicity, the same occurring in severe adult COVID-19 cases (102).

Long- and Post-COVID have been defined by World Health Organization as chronic conditions with persistent, relapsing, remitting, or progressive symptoms occurring three months and years after SARS-CoV-2 infection, respectively (18–20). By the end of 2021, the Institute for Health Metrics and Evaluation (IHME) estimated 3.7% (144.7 million) of individuals developed LC, with 15.1% (22 million) presenting persistent symptoms at 12 months after SARS-CoV-2 infection (103). However, these numbers are likely underestimated, since only in October 2021 WHO introduced a specific 10th revision of the International Classification of Diseases (ICD-10) code, U09.9, designated as “Post COVID-19 condition, unspecified”. Based on that is a challenge specific to LC cases mortality data, mostly because of disparities in definitions, clinical diagnosis inaccuracies, and documentation.

**Figure 3:**
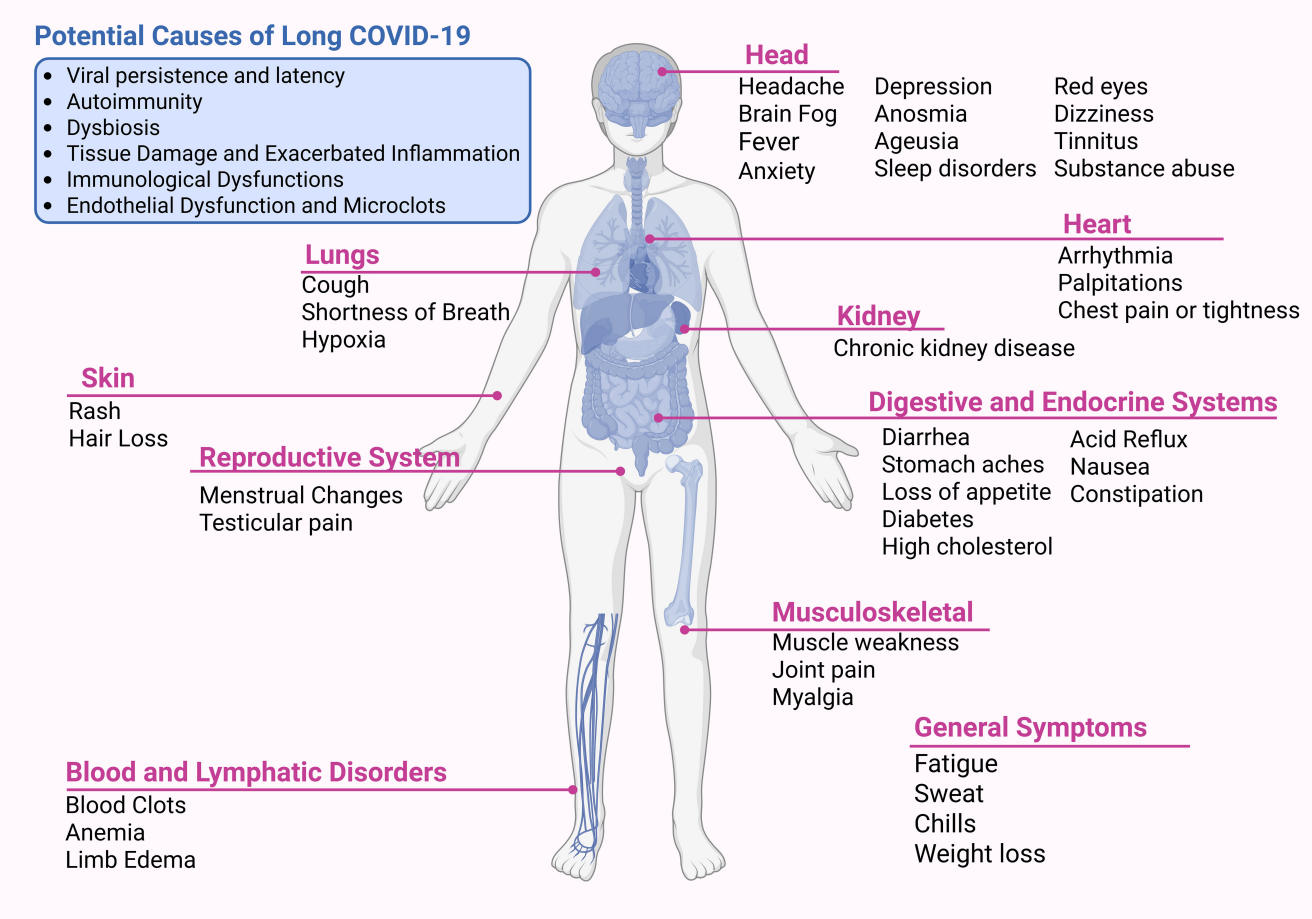
Potential causes and multi-organ manifestations of Long COVID. Long COVID is a heterogeneous condition with proposed mechanisms including viral persistence, autoimmunity, dysbiosis, tissue damage with exacerbated inflammation, immunological dysfunction, and endothelial pathology with microclot formation. Symptoms affect multiple systems, including neurological, cardiovascular, pulmonary, renal, gastrointestinal, musculoskeletal, dermatological, and reproductive, as well as blood and lymphatic disorders. Common symptoms include fatigue, cognitive impairment (“brain fog”), dyspnea, palpitations, sleep disorders, depression, and myalgia. Created using Biorender: https://BioRender.com/w1nodz2.

Over 200 symptoms across multiple organ systems have been reported by individuals with Long COVID, with the most common including persistent fatigue, muscle or joint pain, shortness of breath, headache, difficulty concentrating, memory impairment, and alterations in taste and smell (20) (**Figure 3**). Although growing research provides valuable insights, the underlying causes of LC remain unclear. Several pathogenic mechanisms have been proposed, with four predominant hypotheses encompassing viral persistence, latent viral reactivation, autoimmunity, and chronic inflammation (21). These mechanisms are not mutually exclusive but rather interrelated, forming a continuum in which acute-phase immune and inflammatory disturbances evolve into chronic cellular inflammatory states, leading to tissue injuries and other conditions such as platelet activation, coagulation abnormalities, microclot formation, and impaired gas exchange (104).

Persistent viral presence, antigen reservoirs, and residual spike protein may remain active within body tissues (105), accompanied by a delayed or dysregulated immune response to viral antigens, contributing to the development of Long COVID symptoms. The persistence of SARS-CoV-2 in the body, even after apparent viral neutralization, has been consistently associated with Long COVID manifestations (106, 107), possibly because such viral persistence sustains continuous immune activation, leading to the ongoing production of functional autoantibodies (fAABs) that can affect multiple organs. However, the underlying immunological mechanisms remain poorly understood, particularly considering that viral persistence is not a phenomenon unique to SARS-CoV-2 but has also been described in other chronic or post-viral conditions (108). Associated with persistent immune activation, Epstein-Barr virus viremia and SARS-CoV-2 RNAemia were positive in individuals at diagnosis, showing a reactivation of latent virus anticipating LC patterns (109).

Classical autoimmunity arises from a loss of self-tolerance, which is determined by an individual’s genetic and epigenetic background and further modulated by external factors such as lifestyle and environmental exposures, such as infections (110). During the acute phase, the SARS-CoV-2 virus may trigger autoimmune disease, elevating the levels of autoantibodies. Kreye et al. (2020), observed that a large fraction of antibodies generated following SARS-CoV-2 infection recognize host proteins rather than viral targets, indicating autoimmune reactivity (111). This could be attributed to the fact that SARS-CoV-2 encodes two viral proteases, NSP3 (PL^Pro^) and NSP5 (3CL^Pro^), which may directly contribute to autoimmunity by extensively cleaving host proteins that share consensus cleavage sites with viral polyproteins (112). This process generates thousands of novel peptide fragments (neoantigens) that are presented on MHC molecules and misrecognized as “non-self” by cytotoxic T cells, leading to chronic activation of autoreactive CD8^+^ and CD4^+^ T cells, persistent inflammation, and functional autoantibody production. The resulting neoantigen-driven mechanism further disrupts key immunoregulatory pathways, impair type I and II IFN responses, hinder viral clearance, and promote long-term viral persistence in tissues, ultimately sustaining immune activation and the relapsing symptomatology characteristic of Long COVID (112).

Emerging evidence suggests that several classes of autoantibodies are associated with the occurrence, symptomatology, and severity of Long COVID. In particular, the persistence of antinuclear autoantibodies (ANAs) for up to 12 months post-infection in patients with post-COVID syndrome, overlapping with autoimmune features of lupus (SLE), rheumatoid arthritis, or Sjögren’s syndrome (113). Also, the presence of functional autoantibodies (fAAbs) targeting G protein–coupled receptors (GPCRs), has shown a significant association with symptoms such as dizziness, lack of concentration, postural orthostatic tachycardia syndrome, and the deterioration of pre-existing neurological conditions (114). These autoantibodies have been identified as potential immunological biomarkers, aiding in the diagnosis, prognostic evaluation, and assessment of disease severity in affected individuals, and their therapeutic removal may confer clinical benefits, supporting their role as active contributors to the pathophysiology of Long COVID/post-COVID syndrome (115).

Because of a wide range of symptoms and gaps in the origin of Long COVID, likely influenced by individual differences in neoantigen presentation via MHC molecules (112), the major challenge is about accurate diagnosis. Besides self-reported tests (116), most diagnostic tools for LC are still under development, particularly imaging methods aimed at identifying organ-specific alterations (117–119). Moreover, some studies suggesting LC biomarkers are growing. A recent genome-wide study has proposed an association of FOXP4 with LC. The C allele at rs9367106 was associated with an increased risk of LC but not necessarily as a causal variant. Otherwise, rs9381074 was identified as a causal variant because of its association at the *FOXP4* locus. Higher expression of *FOXP4* in immune and alveolar cells (type 2) also contributes to a role in LC. Although the limitation of the study was the data collected prior to the Omicron wave and widespread vaccination, *FOXP4* can be better investigated for possible contribution as a genetic risk factor to LC (120). Another study focusing on finding a mRNA signature in LC patients, Missailidis et al. (2024), through a transcriptome analysis of peripheral blood mononuclear cells (PBMCs) showed 2 genes downregulated from 70 genes, identified as leukocyte immunoglobulin-like receptors, *LILRB1* and *LILRB2*, with the potential to discriminate all of the LC from recovered patients (121). Still examining the immune profile of PBMCs, Guerrera et al. (2025) showed CD3+ T, CD4+ T, CD8+ T, and Treg cells reduced levels in COVID-19 patients. However, in LC patients all cells were recovered, but the numbers of CD8+ T cells remained low, as well as activation markers on both CD8 T cells and γδ T cells, including the expression of CXCR5 and CCR6, identifying an immunological signature in adaptive immune dysregulation (122). Furthermore, healthy convalescent individuals exhibited higher titers of SARS-CoV-2 neutralizing antibodies compared to those with long COVID. Detailed phenotypic analyses revealed a modest upregulation of co-inhibitory receptors, particularly PD-1 and TIM-3, on SARS-CoV-2 non-spike-specific CD8^+^ T cells in long COVID patients (123). Elevated levels of inflammatory biomarkers, such as IL-6, CRP, and TNF-a, after SARS-CoV-2 infection for one or more months were found to be a potential core set of biomarkers for long COVID, which can be used to manage long COVID patients in clinical practice (124). Recently, Abbasi et al. (2025) identified SARS-CoV-2–derived peptides from the viral replicase polyprotein 1ab (Pp1ab) within serum extracellular vesicles of patients with persistent long COVID symptoms, suggesting that Pp1ab-enriched EVs may serve as a potential biomarker of persistent viral activity and ongoing pathogenesis (125).

Notably, all these efforts to find biomarkers for LC diagnosis and future targeted therapy have many unclear mechanisms. Among promising approaches arises from an ongoing clinical study with the DNA aptamer rovunaptabin, also known as BC007, which neutralizes functional autoantibodies (fAABs) targeting G protein–coupled receptors (GPCRs) (112, 126).

Therefore, patients who developed severe illness from COVID-19 had a greater risk of LC-associated symptoms than non-severe illness (127), although long COVID can develop regardless of the severity of the initial SARS-CoV-2 infection (128). Recent studies have shown an increased risk of Long COVID after repeated infections (129). Although vaccination helps mitigate this risk, the emergence of immune-evasive Omicron variants led to widespread reinfections, underscoring the need for continuous vaccine updates to improve protection against evolving variants of concern. Overall, a cohort study of 2025 demonstrated that less than 2% of LC patients followed up to 3 years after initial infection had resolution of symptoms, but that the COVID-19 vaccination was associated with better outcomes (130). Notably, the vaccination status and variant of SARS-CoV-2 may influence the risk of long COVID and changes in LC symptoms (28, 131, 132).

## 4 COVID-19 vaccine development

**Figure 4:**
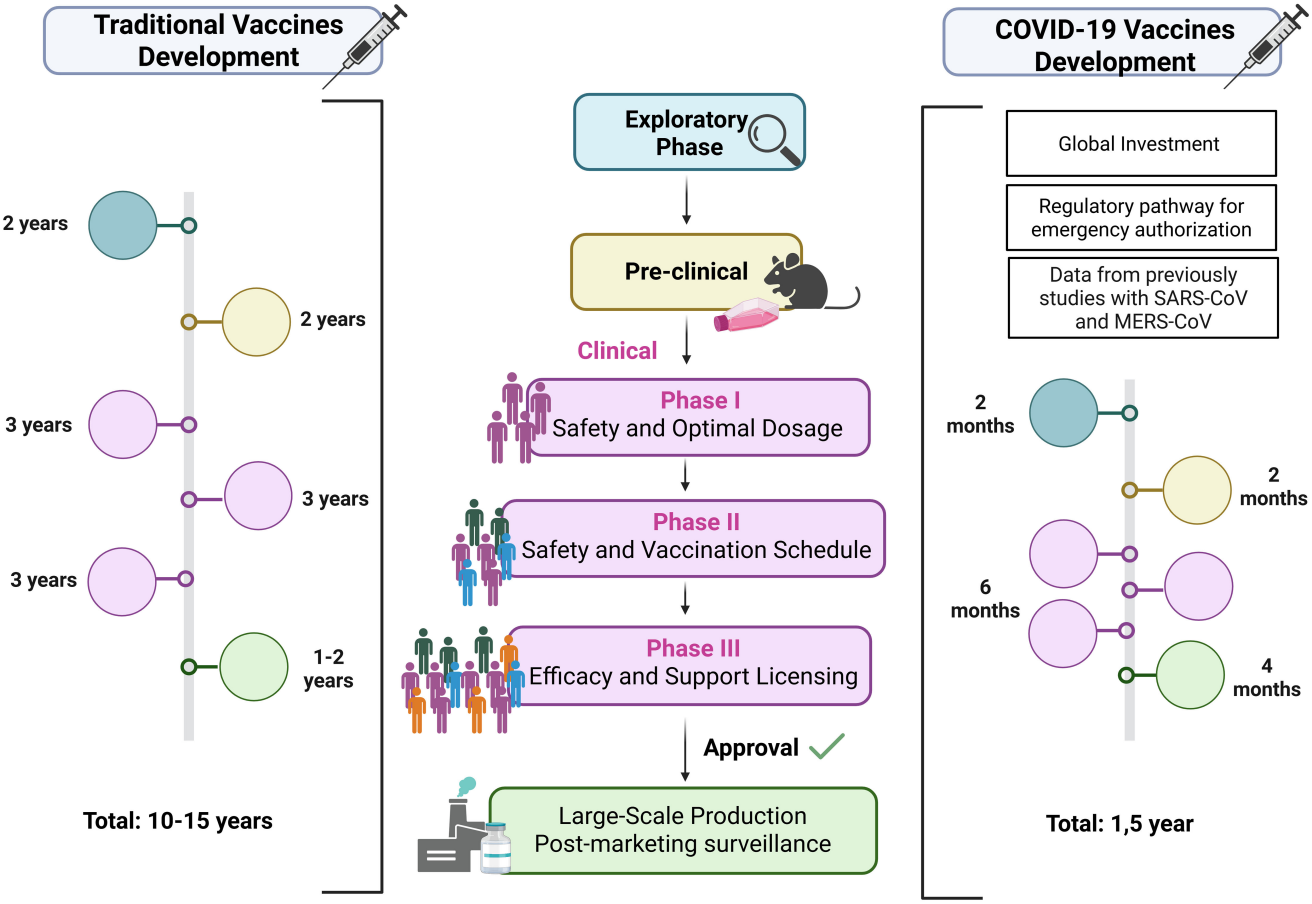
Traditional and accelerated COVID-19 vaccine development timelines. Traditional vaccines take >10 years: exploratory phase (pathogen study, platform selection), preclinical tests (safety, immune response in cells/animals), and sequential clinical trials — Phase I (safety, dose), Phase II (schedule, regimen), Phase III (efficacy, licensing) — followed by long-term surveillance. COVID-19 vaccines were developed in ~1.5 years through global investment, emergency authorization, prior SARS/MERS vaccine data, and adapted production platforms. Exploratory stages were shortened, preclinical and clinical phases ran in parallel, and large-scale manufacturing began before trial completion, enabling rapid rollout once safety and ≥50% efficacy were demonstrated. Created using Biorender: https://BioRender.com/28gba5n.

To rapidly respond to the COVID-19 pandemic, several technological platforms were employed in vaccine development, each with specific advantages and limitations. Some aspects must be considered in choosing the vaccine development platform: technology, time and costs involved in large-scale production, storage conditions, administration route, dose regimen, nature and durability of the immune response, and the possibility of updating vaccines in cases of emergence of variant strains with potential to evade immunity (133, 134) (**Figure 4**). Among the most widely used were inactivated virus, viral vector, and mRNA vaccines, which together accounted for the majority of doses administered globally.

### 4.1 COVID-19 vaccination

Inactivated virus vaccines are based on a long-established approach, historically used for pathogens such as influenza, polio, hepatitis A, rabies, and typhoid (135). They are produced by growing large amounts of the pathogen, followed by particle inactivation using agents such as formaldehyde or β-propiolactone (BPL) (7, 9–136). By presenting the complete viral surface to the immune system, they elicit a broad antibody repertoire but generally limited T cell activation, requiring the addition of adjuvants and administration in multidose regimens (67). During the COVID-19 pandemic, CoronaVac (Sinovac Life Science Co., China) was the most administered inactivated vaccine. Delivered in a two-dose intramuscular regimen, it demonstrated the capacity to elicit robust humoral immune responses and to reduce hospitalizations and deaths (137–140). Regarding the cellular response, a significant percentage of the circulating SARS-CoV-2-specific CD4+ T cells detected after two doses of CoronaVac exhibited a Tfh phenotype, similar to those observed following mRNA vaccination and infection (141).

Viral vector vaccines use recombinant, replication-deficient viruses engineered to express antigens from the target pathogen, inducing strong humoral and cellular immune responses without adjuvants (142). Adenoviruses are most commonly used because they are genetically stable, induce potent CD8^+^ T cell responses, and remain effective in resource-limited settings due to their relative thermostability (142, 143). This innovative technology has been studied in the development of vaccines for various infectious diseases such as HIV, dengue, flu, tuberculosis, malaria, Zika, chikungunya, MERS, among others (134). For COVID-19, ChAdOx1 nCoV-19 (AstraZeneca/University of Oxford) and Ad26.COV2.S (Janssen/Johnson & Johnson) were two of the most widely deployed viral vector vaccines, both of which express the SARS-CoV-2 spike protein. ChAdOx1, based on a chimpanzee adenovirus vector ChAd, was typically administered in a two-dose regimen (138, 144, 145), while Ad26.COV2.S, based on human adenovirus serotype 26, was the only COVID-19 vaccine approved for use as a single-dose primary schedule, facilitating rapid coverage in populations with limited healthcare access (138). Ad26.COV2.S elicits broad humoral and cellular immune responses, which are associated with protective effectiveness against SARS-CoV-2 infection, moderate to severe COVID-19, and COVID-19–related hospitalization and death (146, 147). The ChAdOx1 vaccine induces a Th1-biased response characterized by IFN-γ and TNF-α secretion by CD4^+^ T cells, predominant IgG1/IgG3 production, and CD8^+^ T cells with cytotoxic potential (148), and has demonstrated efficacy against symptomatic infection by the ancestral SARS-CoV-2 strain (149).

mRNA vaccines, a more recent platform, enabled unprecedented speed in COVID-19 vaccine development thanks to their capacity for rapid design and large-scale production without the need to manipulate live pathogens (150–152). These vaccines deliver messenger RNA encoding the spike protein into host cells via lipid nanoparticles (LNPs), enabling intracellular antigen expression that mimics natural infection and elicits both humoral and cellular immune responses (150–152). Modifications such as nucleoside analog incorporation and optimized purification increase stability and translation efficiency (153, 154). For COVID-19, BNT162b2 (Pfizer/BioNTech) and mRNA-1273 (Moderna) were the first mRNA vaccines authorized for human use, marking a milestone in vaccinology. Both mRNA-based vaccines targeted the prefusion full-length spike protein and were administered as two-dose regimens, although they differ in the mRNA content, the LNP composition and the interval between priming and boosting doses (155–158). Early randomized trials demonstrated that both vaccines were highly effective in preventing symptomatic COVID-19 (156, 158), and may offer increased immunogenicity in immunocompromised (IC) patients, particularly with the higher-dose mRNA-1273 vaccine, which is associated with higher seroconversion rates, greater total anti-spike antibody titers, and elevated neutralizing antibody titers and cellular immune responses (159), the latter being linked to protection against initial SARS-CoV-2 infection, viral clearance, and potentially essential for long-lasting immunity (160).

Together, these three vaccine platforms, based on different technological principles, formed the backbone of the global immunization effort against COVID-19. Some were later supplemented with booster doses to counter waning immunity and updated to enhance protection against variants of concern, helping to maintain vaccine effectiveness throughout the pandemic.

### 4.2 Variants of concern and vaccine reformulation

#### 4.2.1 Emergence of variants of concern

SARS-CoV-2, an RNA virus, accumulates mutations more frequently than DNA viruses. The accumulation is largely due to the RNA-dependent RNA polymerase (RdRp) enzyme, which is inherently error-prone during the virus’s replication process and lacks the ability to correct these errors. These mutations can alter amino acid sequences and protein function, driving viral evolution, increasing pathogenicity, and potentially compromising immune responses. Asymptomatic transmission further facilitates viral spread and the emergence of selective mutations that enhance replication, transmissibility, or immune evasion tend to become dominant (161). Of particular concern are mutations in the Spike protein, especially in the receptor-binding domain (RBD), which can increase ACE2 affinity or promote antibody escape. Variants with significant impacts on disease severity or reduced vaccine and treatment efficacy are classified as variants of concern (VOCs) (94).

Throughout the COVID-19 pandemic, several VOCs were originated: Alpha or B.1.1.7 (UK-originated variant), Beta or B.1.351 (South African-originated variant), Gamma or P.1 (a Brazilian variant), Delta or B.1.617 (an Indian variant) and Omicron or B.1.1.529 (South Africa and Botswana- first detection) (162–165). Among them, the Omicron and its subvariants were highlighted, a lineage with up to 60 mutations, 38 of which are in the Spike protein and at least 12 in the RBD domain. Most of these mutations are known to affect transmissibility, immune evasion and a higher risk of reinfection (166). Among the mutations found in this SARS-CoV-2 lineage, D614G and P681H are linked to greater infectivity and stability of the viral particle (167, 168). While N501Y, T478K, Q493R, and H655Y increase the affinity of SARS-CoV-2 for the ACE2 receptor, increasing its transmissibility (168). The K417N, E484A, Y505H, N440K, and G496S mutations favor immune escape via neutralizing antibodies generated during vaccination or natural infection, as well as alter the sensitivity to neutralization by monoclonal antibodies or sera from convalescent patients, compromising the effectiveness of treatments (169). However, clinical pathogenicity in Omicron is reduced when compared to other VOCs (170), which is believed to be associated with TMPRSS2-independent viral fusion and replication (171, 172).

The newly emerged variants have raised concerns about the immunity conferred by COVID-19 vaccines, particularly in mRNA vaccines and vector vaccines, which were designed to express the spike glycoprotein based on the reference sequence. Studies found neutralizing antibody evasion by Omicron sublineages using sera from individuals vaccinated with both vaccine strategies (173, 174), yet neutralizing capacity was partially restored by vaccine boosters (175–177). Nevertheless, antibodies produced by a triple homologous/heterologous vaccination regimen or by hybrid immunity with two doses of the vaccine resulted in greater neutralizing capacity against the Omicron variant (178, 179). Although neutralizing antibody titers represent only one component of the vaccine-induced response, it is crucial to assess the effectiveness of vaccines in response to variants and, if necessary, adapt them. However, vaccines based on disease-causing pathogens are complex to update in cases of the emergence of viral variants that have undergone mutations and escaped from the immune system. A study observed that the variant of concern P.1 Gamma can escape the neutralization of antibodies from convalescent patients and individuals previously immunized with the inactivated vaccine (CoronaVac) and that this neutralization capacity is six times smaller when compared to the original Wuhan lineage (180), highlighting the challenge of immune escape in this platform.

Although these mutations provided greater escape from neutralizing antibodies and reduction of B memory cells, the other arm of the adaptive immune response generated by vaccines, memory CD4+ and CD8+ T cells, has also been explored. It has been observed that T-cell-mediated immunity was well maintained for long periods after vaccination with the monovalents Wuhan vaccines and confers cross-protection to SARS-CoV-2 variants, which would explain the lack of severe cases and deaths in reinfected and vaccinated individuals (181, 182). This even applies to the Omicron variant, with preservation of the epitopes recognized by T cells with an average > 80% for Omicron compared to the other VOCs (183, 184).

#### 4.2.2 Boosters and vaccine adaptation

Due to the rapid decline in neutralizing antibody levels 6 months after the second dose and the emergence of VOCs such as Delta, which are more transmissible and cause serious diseases than others variants of the virus (185), the application of booster doses of the vaccine was globally recommended, mainly for immunocompromised individuals, those with comorbidities and healthcare professionals (186). The boosters were able to restore neutralizing antibody titers.

To address the ongoing evolution of SARS-CoV-2, particularly the emergence of Omicron and its subvariants, some vaccines were reformulated and administered as boosters to broaden immune protection. These updates mainly involved mRNA vaccines due to their adaptability, leading to the development and authorization in 2022 of bivalent formulations, such as Comirnaty (Pfizer-BioNTech) and Spikevax (Moderna), which combine mRNAs encoding the original Wuhan strain with those targeting the Omicron BA.1 and BA.4/5 variants (187, 188). Next, monovalent vaccines targeting the Spike protein of subvariants XBB.1.5 and KP-2 have also been approved by the same companies (15, 189).

Souza et al. (2024) reported that individuals who received a single dose of a bivalent vaccine exhibited significantly higher neutralizing antibody levels against various SARS-CoV-2 variants compared to those who received three or four monovalent boosters based on the B.1 lineage (176). Similar results were found by Scheaffer et al. (2022) in mouse models, showing broader neutralization of Omicron variants (177). However, repeated mRNA vaccination against COVID-19 was associated with IgG4 class switching, which reduces Fc-dependent antibody effector functions and diminishes NK cell activation by S1-specific antibodies, potentially weakening the vaccine-induced immune response (190, 191). Regarding T cell-mediated immunity, it remained stable after two doses of the monovalent Wuhan-based vaccine and was not significantly enhanced by further booster doses or Omicron breakthrough infections (82), suggesting that T cell responses are more antigenically resilient to Spike mutations and continue to contribute to protection against severe COVID-19. Notably, SARS-CoV-2-specific T cells induced by booster vaccines remained active and functional for at least one year post-immunization (82).

## 5 Impact of vaccination on long- and post-COVID syndromes

### 5.1 Immunological and virological mechanisms of protection

**Figure 5:**
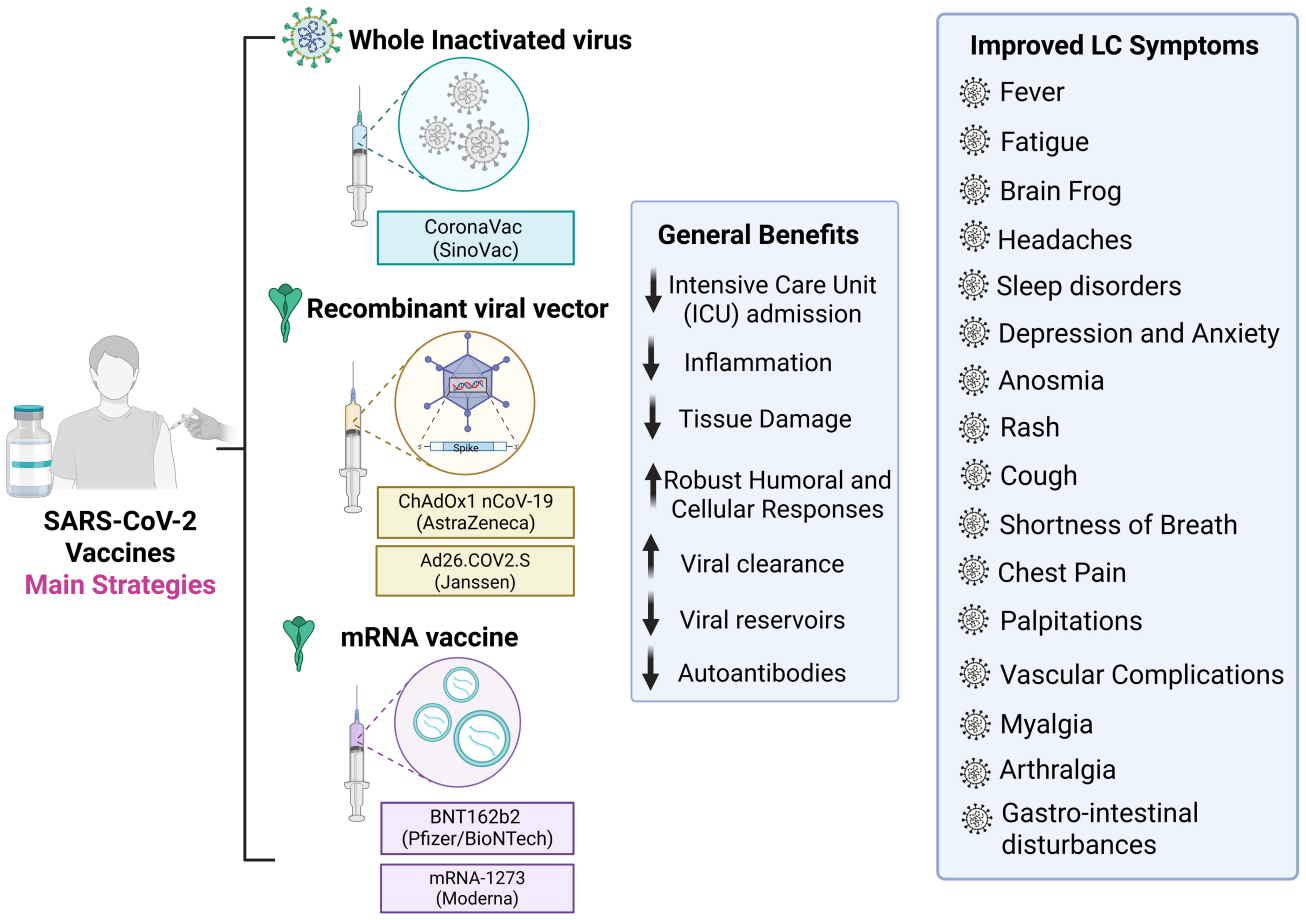
Vaccine strategies against SARS-CoV-2 and potential benefits for Long COVID prevention and mitigation. The hallmark of the COVID-19 pandemic was the diverse range of technological platforms employed in the development of vaccines against SARS-CoV-2. Among the most widely used were inactivated virus, viral vector, and mRNA vaccines, which together accounted for the majority of doses administered globally. Vaccination reduces severe COVID-19 outcomes, inflammation, tissue damage, and viral persistence, while promoting robust humoral and cellular immune responses and lowering autoantibody levels. Post-vaccination improvements in Long COVID symptoms include reduced fatigue, brain fog, headaches, sleep disturbances, depression, anxiety, anosmia, cough, dyspnea, chest pain, palpitations, vascular complications, myalgia, arthralgia, and gastrointestinal disturbances. Created using Biorender: https://BioRender.com/o1um17d.

Vaccination against SARS-CoV-2 does not completely prevent long COVID (LC) but significantly reduces its incidence and symptom burden. Multiple studies have demonstrated lower frequencies of fatigue, fever, cough, dyspnea, anxiety, depression, memory dysfunction, and brain fog among vaccinated individuals compared with unvaccinated peers, with effects persisting for up to 18 months (192–194). Breakthrough infections among vaccinated individuals are also associated with lower LC risk, particularly for coagulation and pulmonary sequelae (27). Notably, protection increases with acute disease severity, being strongest among hospitalized and ICU-treated patients (195) (**Figure 5**). The underlying mechanisms for these benefits are multifactorial and involve both immunological and virological processes.

A primary protective mechanism of COVID-19 vaccination involves attenuation of the severity of the disease in the acute phase, which has been consistently associated with a higher risk of post-acute sequelae, due to intense immune activation, extensive tissue damage, and prolonged hospitalization. Vaccinated individuals typically experience a milder course of acute illness, leading to reduced tissue damage and a lower risk of long-term complications (27, 192, 193). In a pediatric prospective cohort, reduced organ damage following vaccination was proposed as a key physiological explanation for the decreased incidence of long COVID (195). Notably, a higher viral burden during the acute phase is strongly associated with an increased risk of persistent symptoms (196–199), indicating that early immune priming through vaccination may help interrupt this pathological trajectory (27).

Vaccinated individuals tend to exhibit more regulated immune responses during acute SARS-CoV-2 infection, characterized by a lower incidence of cytokine storms and reduced prevalence of autoantibody formation (192). Studies have consistently reported that COVID-19 vaccination is associated with a downregulation of pro-inflammatory cytokines such as IL-1, IL-6, and TNF-α, which are key mediators involved in chronic inflammation and fibrotic tissue damage (192, 200, 201). In addition, elevated plasma levels of soluble CD40 ligand (sCD40L) have been observed in moderate to severe COVID-19, associated with a platelet-driven prothrombotic profile (202). Notably, sCD40L concentrations were significantly reduced following vaccination (203). Moreover, vaccinated individuals show fewer symptoms linked to enhanced inflammatory responses, such as headaches, joint pain, and dysregulated hypertension. These symptoms were significantly more frequent in unvaccinated patients, suggesting that stronger inflammatory reactions in the absence of vaccination may underlie these clinical differences (192). From an immunological standpoint, these protective effects reflect the benefits of early immune priming: vaccinated immune systems are better equipped to control viral replication and mount proportionate responses, thereby reducing collateral tissue damage and systemic inflammation during acute infection (27, 204). These findings support the immunological theory that primed immune systems respond more efficiently and with less dysregulation during acute SARS-CoV-2 infection, thereby reducing downstream post-acute sequelae.

Faster viral clearance and the prevention of viral reservoir establishment have emerged as prominent immunological mechanism candidates by which COVID-19 vaccination may influence the development and severity of LC (27, 28, 203, 204). Persistent SARS-CoV-2 RNA or antigens are thought to drive chronic immune activation and inflammation, dysbiosis, and coagulation abnormalities, contributing to key long COVID symptoms, including fatigue, increased risk of thrombosis, cognitive dysfunction, and myalgia (21, 205). Supporting this hypothesis, SARS-CoV-2 components have been detected months after infection in immune-privileged tissues, such as the intestine, skin, liver, and lungs (206–208). Among viral proteins, the persistence of spike protein in various tissues may contribute to prolonged neurological symptoms observed in long COVID-19 (104, 105, 209, 210). Furthermore, circulating spike antigen has been identified in approximately 60% of PCC patients up to 12 months post-infection (104), and spike S1 protein has been detected within monocytes as late as 15 months after acute infection (209). SARS-CoV-2 Spike protein plays a pivotal role in COVID-19 pathogenesis and is the main target for vaccine development. Spike protein can also aberrantly activate the innate immune Toll-like receptor 4 (57, 211–213). Notably, S1 may persist in plasma via extracellular vesicles (EV) (214) and has been shown to associate with LPS, especially in Delta variant infections, contributing to inflammation (215). S1 also stimulates pro-inflammatory cytokine production, and its presence in monocytes and granulocytes (209) suggests these cells may sustain chronic inflammation in post COVID syndrome (198). These findings reinforce the role of persistent viral material in perpetuating immune imbalance and long COVID symptoms (16, 216).

A pre-existing immune response induced by vaccination can prevent the establishment of viral reservoirs, thereby reducing the risk of long COVID (205). This protection is primarily mediated by the rapid induction of neutralizing antibodies and virus-specific T cell responses, which limit viral replication and facilitate early viral clearance. SARS-CoV-2 mRNA vaccines elicit transient cytokine responses associated with robust spike-specific antibody production, including in previously infected individuals (200). Recent studies revealed that healthy convalescents displayed higher neutralizing activity against SARS-CoV-2 than individuals with long COVID despite comparable anti-Spike IgG titers, suggesting qualitative differences in humoral immunity (123). This observation indicates that vaccination not only enhances immune magnitude but also optimizes functional breadth, leading to more efficient viral control and reduced risk of chronic antigen persistence.

However, the emergence of Omicron and its heavily mutated subvariants posed a major challenge, as extensive conformational alterations increased both transmissibility and immune evasion. Although Omicron generally causes less aggressive acute disease compared to previous VOCs (170), these sublineages remain globally predominant and have resulted in frequent reinfections, even among vaccinated individuals. Despite its increased transmissibility and number of reinfections, studies have shown that the Omicron variant presents a low risk of developing LC when compared to previous SARS-CoV-2 variants (217). On the other hand, repeated exposure to Omicron variants may promote sustained immune activation and the generation of autoantibodies, potentially increasing the risk of developing persistent symptoms consistent with Long COVID (129, 218). Thus, continued vaccination efforts with updated formulations targeting currently circulating variants remain essential to prevent reinfections and mitigate the long-term consequences of SARS-CoV-2 infection (112, 219).

Autoimmunity has been proposed as a key mechanism underlying long COVID, either through molecular mimicry or by the generation of neoantigens. Antibodies recognizing SARS-CoV-2 spike protein cross-react with several human tissue antigens, especially neurological (220), were detected in patients with severe acute SARS-CoV-2 infection with reactivity to pro-inflammatory factors (221), can even contribute to cardiovascular inflammation via atherosclerotic plaque formation (222). Beyond molecular mimicry, viral proteases (PL^pro^ and 3CL^pro^) may contribute to autoimmunity by aberrantly cleaving host proteins, generating neoantigens that are presented by MHC molecules and misrecognized as “non-self.” This process can trigger sustained activation of autoreactive T cells, persistent inflammation, and autoantibody production, reinforcing the autoimmune mechanisms implicated in post COVID (112).

Vaccination may prevent the emergence of autoantibodies by reducing the risk of reinfections, promoting faster viral clearance, and modulating the immune response, thereby diminishing the production of inflammatory cytokines and chemokines and/or reprogramming pathogenic lymphocytes (200). Notarte et al. (2022) compiled evidence supporting the notion that pre-infection vaccination may reduce autoimmunity-related long COVID manifestations (28). Although inflammation impairs immunocompetence, it is possible that vaccination in individuals with pre-existing long COVID symptoms promotes anti-Spike IgG responses which may have helped lower a persistent viral burden and reduce titers of autoantibodies (223).

In immunocompromised populations, the effectiveness of vaccination against long COVID remains inconclusive. Studies in people living with human immunodeficiency virus (HIV) infection (PLHIV) (224) and those with common variable immunodeficiency (CVID) (225), which consistently exhibit higher rates of long COVID compared to community-based cohorts, reported a lower long COVID prevalence among vaccinated individuals. However, these associations were not statistically significant, largely due to limited sample sizes and methodological limitations. These findings underscore both the benefits and the limitations of vaccination in preventing long COVID, especially in vulnerable populations.

### 5.2 Vaccine platforms and dosage

Large cohort studies have consistently demonstrated the significant efficacy of vaccination in reducing the severity, duration of long COVID symptoms, and overall hospitalization rates. In a multicentric analysis across the UK, Spain, and Estonia, pre-infection vaccination was associated with a markedly lower risk of long COVID (226). Ranucci et al. (2023) found that vaccinated hospitalized COVID-19 patients had significantly lower rates of major physical and neuropsychological symptoms at 12 and 18 months post-infection (MPS: 52% vs. 91.7% in unvaccinated; MNS: 24% vs. 93.8%) (193). Similarly, Babicki et al. (2023) and Ioannou et al. (2022) reported reduced incidence of symptoms such as headache, joint pain, and documented long COVID diagnoses among fully vaccinated individuals (192, 227). Collectively, these findings support the hypothesis that vaccination mitigates long COVID primarily through accelerated viral clearance and prevention of persistent antigenic stimulation. However, a better understanding of the similarities and differences between vaccine platforms and dosing regimens is needed, not only in preventing the development of long COVID, but also in evaluating their impact on individuals with pre-existing long COVID symptoms.

Multiple studies have reported a slightly stronger preventive effect of mRNA vaccines compared to adenoviral vector vaccines in reducing the risk of developing long COVID symptoms. Català et al. (2024) found that administration of any first dose of COVID-19 vaccine (ChAdOx1, BNT162b2, Ad26.COV2.S or mRNA-1273) was associated with a reduced risk of long COVID, with a slightly greater preventive effect observed for BNT162b2 compared to ChAdOx1 (226). Similarly, other studies have shown a more pronounced reduction in long COVID symptoms among recipients of mRNA vaccines (BNT162b2 and mRNA-1273) relative to those who received adenoviral vaccines (Ad26.COV2.S) (23, 27, 131, 228). Interestingly, a greater improvement in symptoms observed among individuals who received mRNA vaccines compared to those who received adenoviral vector vaccines was more pronounced in key long COVID symptoms such as fatigue, brain fog, and myalgia (229). This difference in vaccine efficacy has been attributed, in part, to the higher effectiveness of mRNA vaccines (particularly BNT162b2) in preventing SARS-CoV-2 infection compared to adenoviral vaccines such as ChAdOx1 (149, 158), as well as the greater protection mRNA vaccines appear to offer against severe COVID-19 illness (28, 228).

It is important to relate that adenoviral vector vaccine ChAdOx1, was associated with rare thrombotic events linked to endothelial inflammation and microvascular injury, mainly observed in young adults (22–49 years) with elevated D-dimer and CRP levels (230–232). Although these cases are uncommon, they have been characterized as vaccine-induced immune thrombotic thrombocytopenia (VITT), resulting from pathogenic immune complexes that activate platelets and leukocytes, leading to thrombosis and thrombocytopenia (233). These findings highlight overlapping mechanisms of vascular inflammation, coagulation, and immune activation in both vaccine-induced thrombosis and long COVID.

Although some studies have shown that even a single dose may be sufficient to reduce the prevalence and severity of long COVID symptoms, two doses are likely more effective (131, 132). Ioannou et al., 2022 demonstrated that only individuals with two doses of mRNA COVID-19 vaccine were protected from long COVID diagnosis, while one dose was insufficient (227). Furthermore, evidence supports a dose-dependent protective effect of COVID-19 vaccination against long COVID, regardless of the vaccine platform used. A study involving over 500,000 adults reported a 21% risk reduction after one dose, 59% after two doses, and 73% after three or more doses compared to unvaccinated individuals (234). Similarly, Marra et al. (2023) found that, among individuals without prior SARS-CoV-2 infection, two doses conferred 37% protection and three doses up to 69% effectiveness against the development of long COVID (235).

However, if autoimmunity contributes to the pathophysiology of long COVID, vaccine-induced expansion of autoreactive clones could, in rare cases, transiently exacerbate symptoms through heightened immune activation and antibody production, as suggested by isolated reports (236). Pediatric registry data noted only slight increases in autoimmune diagnoses post-vaccination, but the absolute risk remained low (237). Korner et al. (2023) identified significantly elevated levels of IgG3 and IgG4 subclasses in long COVID patients with concomitant Myalgic Encephalomyelitis/Chronic Fatigue Syndrome (ME/CFS), suggesting a role in the immunopathology of long COVID (238). Although little is known about virus-specific IgG4 antibody responses in controlling viral infections, evidence has suggested a pathogenic role of IgG4 in autoimmune diseases (239). This class switching toward IgG4 has raised considerable interest, as high IgG4 levels were previously observed in the context of HIV vaccine trials, where vaccine-induced IgG4 responses impaired Fc-mediated effector functions such as antibody-dependent cellular cytotoxicity (ADCC) and phagocytosis (ADCP), thereby questioning their functional implications (240, 241). Furthermore, It has been reported that repeated immunization of naïve individuals with the mRNA vaccines increased the proportion of the IgG4 subclass over time compared with individuals who received AZD1222 homologous vaccination (242, 243), showing that vaccination with mRNA-based vaccines caused a shift in anti-spike antibody repertoire toward IgG4 subclass.

Besides mRNA and adenovirus-based vaccines, a study suggested that receiving three doses of the inactivated (SinoVac CoronaVac) vaccine was associated with significantly lower odds of reporting any LC symptoms compared to receiving two doses. Moreover, these protective effects were similar to those observed with three doses of an mRNA vaccine (Pfizer-BioNTech) (244).

Since it is widely known that vaccine-induced immunogenicity declines over time, combined with the emergence of VOCs, the role of booster doses and updated seasonal vaccines, and their impact on the development of LC, must be carefully assessed. It has been reported that the prevalence of LC during the Omicron wave was about half that of the Delta wave (131), with vaccinated individuals having shorter symptom duration with Omicron (6.87 days) than with Delta (8.89 days), further reduced with boosters (4.4 vs. 7.7 days) (192). Notably, booster vaccination appeared to reduce the risk of developing long-term autoimmune complications after SARS-CoV-2 Delta and Omicron BA.1 or BA.2 variant infection, highlighting its potential protective effect (245). Also, the UK’s Health Security Agency found that LC was 50% less common in double-vaccinated individuals infected with Omicron BA.1 compared to those with Delta, although this was not observed in triple-vaccinated individuals (246). In a study estimating the cumulative incidence of severe post-COVID sequelae one year after infection, a 5.23% decline was observed during the Omicron era compared to the combined pre-Delta and Delta eras. Of this reduction, 28.11% was attributed to changes in the virus and other temporal factors, while 71.89% was attributed to vaccination (204). Although vaccine effectiveness varied with emerging variants, suggesting higher LC incidence during Delta infection wave compared to Omicron, a study showed these differences were not significant after adjusting for sociodemographics, clinical characteristics, and vaccination status (130). Furthermore, the higher transmissibility and immune escape capacity of Omicron led to recurrent infections even among vaccinated individuals, thereby maintaining a residual risk of developing Long COVID, although generally associated with milder acute disease compared with previous VOCs. This suggests that the variation in LC symptoms between variants may be driven not solely by the biological traits of the variants.

While the potential benefits of vaccination in preventing long COVID have been widely evaluated, an increasing number of studies are now assessing its impact on individuals with pre-existing long COVID symptoms (106, 203). Grady et al. (2024) offer preliminary indications of biomarkers that may help predict how people with Long COVID respond to vaccines (22). Although elevated levels of soluble IL-6 receptor (sIL-6R) were associated with clinical improvement, individuals with persistently high levels of IFN-β and ciliary neurotrophic factor (CNTF) did not show any improvement following vaccination. Elevated interferon signaling in individuals who did not improve post-vaccination may reflect persistent immune activation due to ongoing SARS-CoV-2 infection and vaccine failure to clear viral reservoirs or that non-SARS-CoV-2 mechanisms are driving the condition, such as reactivation of latent viruses like EBV, or autoimmune processes. Alternatively, the IFN signature may represent sustained immune dysregulation rather than active infection (22).

## 6 Conclusions

Although we adopted a nonsystematic review strategy, the points raised here reinforce that the current body of evidence available in the literature indicates that COVID-19 vaccination plays a multifaceted role in mitigating the risk of Long and Post-COVID syndromes. By inducing robust humoral and cellular immunity, vaccines limit viral replication and prevent the establishment of persistent viral reservoirs, which are hypothesized to drive chronic symptoms. Multiple studies have found evidence that COVID-19 vaccination before SARS-CoV-2 natural infection may reduce the risk of long COVID and in breakthrough infections, it consistently reduces the incidence of important physical and neuropsychological symptoms (132, 193, 247).

Furthermore, given that recurrent infections driven by the widespread circulation of immune-evasive variants can promote persistent immune activation and increase the likelihood of autoimmune manifestations, vaccine boosters and reformulated vaccines are essential to reestablish and sustain humoral immune responses against emerging SARS-CoV-2 variants, thereby preventing reinfections and mitigating the long-term consequences of infection (82). Other Infections with Chronic Sequelae and the Role of Vaccination Parallels with other post-viral syndromes reinforce the rationale for COVID-19 vaccination as a means of reducing long-term sequelae. Infections such as EBV, influenza, Zika, and human papillomavirus (HPV) are known to trigger persistent symptoms or autoimmune complications. Vaccines against these pathogens, including HPV and varicella zoster, reduce post-infectious complications like Guillain-Barré and postherpetic neuralgia (248–251).

Despite these consistent observations supporting the protective role of vaccination against long COVID, several questions remain unresolved. While the benefits of immunization are well established in the general adult population, gaps remain regarding immunocompromised individuals, the elderly, and children. Continued research using harmonized definitions and longitudinal designs is essential to refine our understanding and to outline public health strategies for future pandemics. Furthermore, the complexity of immune responses, the emergence of immune-evasive variants, the heterogeneity of study designs, and the lack of a standardized definition of long COVID and post COVID syndromes across studies contribute to ongoing uncertainty about the magnitude and consistency of vaccine-mediated protection. Among the difficulties of reviewing the effects of vaccination in the context of long COVID are the different approaches regarding the quantity and duration of post-infection symptoms and the subjectivity in patient classification based on self-report rather than detection of LC biomarkers (17).

Other points to be addressed are gaps in the investigation of the protective potential of inactivated vaccines in the long COVID context, which challenge our immune system with a larger repertoire of viral antigens. Some research groups argue that a more effective vaccination strategy would be based on a heterologous vaccination regimen rather than a homologous regimen, in which an initial booster with an inactivated vaccine followed by a booster with an mRNA vaccine would increase the concentration and antibody response (245, 252). Furthermore, global vaccination faces significant inequalities, with disparities in access and coverage between and within countries, primarily affecting vulnerable and low-income populations. In addition, some studies present conflicting data regarding the benefits and effectiveness of vaccination, such as post-vaccine symptom worsening (236), the generation of autoantibodies (253) and ineffectiveness of the booster dose attributed to immunological imprinting (254).

Therefore, more in-depth studies addressing these gaps are essential to better understand the role of immunization in preventing chronic COVID-19 conditions. In parallel, understanding the underlying causes of post-vaccine symptoms, and determining whether early intervention can prevent long-term complications, may be essential for developing safer, more effective vaccines and for shedding light on the biological mechanisms of Long Covid.
